# Temporal and spatial tuning of optical constants in praseodymium doped ceria by electrochemical means

**DOI:** 10.1515/nanoph-2022-0079

**Published:** 2022-06-13

**Authors:** Dmitri Kalaev, Han Gil Seo, Harry L. Tuller

**Affiliations:** Department of Materials Science and Engineering, Massachusetts Institute of Technology, Cambridge, MA, 02139, USA

**Keywords:** active optical tuning, electrochemical titration, oxygen vacancies, praseodymium doped ceria

## Abstract

Temporal and spatial tuning of the refractive index of optical thin films is desired for flat optics applications. The redistribution of mobile ions in mixed ionic-electronic conductors (MIEC) has been demonstrated to serve as a viable means for achieving optical tuning down to the nanoscale. Here we studied the dynamic range of the optical tuning achievable in the refractive index, in the MIEC oxide – Pr_*x*_Ce_1−*x*_O_2−*δ*_ (PCO), for *x* = 0.1, 0.2 and 0.4, at 500 °C, by *in-situ* spectrophotometry. Significant increases in the modulation of both the imaginary and real optical constants in the visible and the adjacent spectra were obtained for increased doping levels. Device employing an electrochemical titration method was implemented to modulate the oxygen concentration, and thereby the optical transmission of PCO. Incorporation of a patterned top electrode allowed for the demonstration of spatial control of PCO thin film properties by *in-situ* video imaging of the optical switching process. The electrochemically induced optical state is shown to remain non-volatile upon quenching the device to room temperature under applied bias.

## 1 Introduction

Actively tunable optical devices require materials with adjustable optical properties e.g. phase change materials (PCMs) [1–4], liquid crystals [5–8], metal hydrides [9, 10], metal-insulator phase transition materials (e.g. VO_2_) [11–13], electrochromic polymers [14] and other examples [15–17]. Another option for achieving tunability in optical materials is via the use of mixed ionic-electronic conductors (MIECs) that exhibit electrochromic properties upon electric field driven cation (e.g. H^+^, Li^+^) intercalation. A prime example is the use of WO_3_ [18] and other oxides, for the implementation of light modulators [19, 20], dynamic color displays [21–23] and reprogrammable metasurfaces [24]. There is ongoing search for new tunable optical materials that rely on other physical principles and potentially provide complementary functionality to existing options. The authors previously proposed rare earth and transition MIEC metal oxides, with oxygen ions as the mobile species, as promising tunable optical materials, amenable to changes in their optical properties within the NIR-VIS-UV spectrum [24]. For example, initially transparent, wide band gap CeO_2_ (*E*_g_ > 3 eV) becomes optically absorbing and tunable when the Ce cations are partially replaced by Pr [25, 26], which has a mixed 4+/3+ valency in the Pr_*x*_Ce_1−*x*_O_2−*δ*_ (PCO) solid solution system. Only Pr in its most oxidized state (Pr^4+^) is optically absorbing. Successively reducing Pr^4+^ to Pr^3+^, via generation of positively charged oxygen vacancies and compensating electrons that localize on the Pr ions within the band gap, enables one to tune the optical absorption of PCO. The Pr dopant readily undergoes a redox reaction, under modest oxygen activity and temperature conditions, that enables modulation of the optically absorbing concentration over the whole doping range, that can reach as high as 40% in PCO. That change is comparable to the cation insertion levels achieved in intercalation materials [21]. However, electronic charges associated with intercalated cations tend to delocalize at high doping concentrations [27], causing significant unwanted optical losses, e.g. for implementation of phase optical actuators [28], especially in the NIR [19, 24]. Thermally tunable materials, e.g. PCM and VO_2_, that change to a metallic phase at a given transition temperature suffer from the excessive optical losses as well. While for PCO, strong electron localization, at energy levels associated with the observed optical transitions, persists even at very high concentrations, keeping the unwanted optical losses in the NIR region due to the free charge carriers very low. Further, PCM and VO_2_ materials are largely used for switching optical devices [12, 29] as they lack the ability to achieve continuous optical tuning due to abrupt changes in optical properties associated with thermally induced phase transitions. On the other hand, PCO enables gradual fine tuning of the refractive index by modifying the concentration of optically absorbing ions, providing a versatile material system for implementation of tunable optical components. Recent studies on dynamic color displays report relatively slow switching times, from minutes to hours, for metal hydrides [9, 30] and cation intercalation metal oxides [19, 21, 31]. Faster tuning of optical properties can be achieved in PCO based devices by utilizing thermally enhanced oxygen vacancy transport (activation energy of ∼0.8 eV) [32] at elevated temperatures, that can be also exploited to achieve non-volatility, i.e. long retention times, of the tuned state near room temperature. For fast optical tuning, the temperature of the whole device can be raised [29] as done in this study; for applications that require spatial control of the refractive index, e.g. pixels in a dynamic color display, they can be controlled in a local manner, e.g. by joule heating [2]. In this study switching times on the seconds scale were shown at 500 °C, with further improvements possible by scaling down device dimensions or by applying higher electrical fields [25].

**Figure 1:**
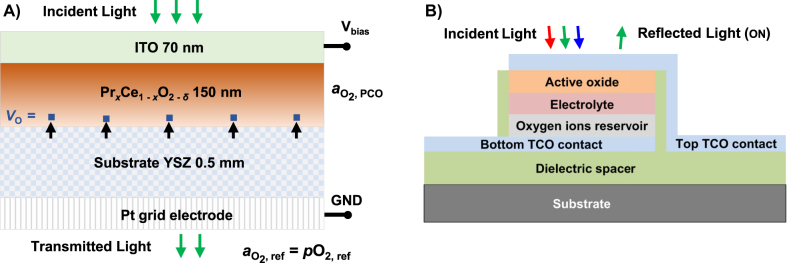
Electrochemical optical modulation device structures. (A) Cross section of four-layered stack of ITO/PCO/YSZ/Pt used to study the active optical tuning of PCO thin films with different compositions. Optical tuning, centered in the visible spectrum, is achieved via electrochemical titration of oxygen through the YSZ electrolyte (oxygen moves in YSZ and PCO via oxygen vacancies–*V*_O_) into and out of the actively tunable PCO layer (see text for details). (B) Conceptual design (cross section) of proposed electrochemically controlled all-solid-state one color device (pixel) for a non-volatile reflective display. The actively tunable PCO layer enables modulation of the device reflectance via the same mechanism as explored in this study in the transmission device.

We previously studied optical tuning in PCO by *in-situ* transmission spectrophotometry and were able to demonstrate that both the real (*n*_r_) and imaginary (*k*) parts of the PCO refractive index could be modified by varying its oxygen stoichiometry, *δ*, either by chemical means or locally by application of an electric field [25]. In these approaches, gradual changes in *n*_r_ and *k* of the PCO refractive index were achieved, reaching as high as 0.1 (∼4% change) and 0.1 (nearly three orders of magnitude change), respectively. Here, we extend such studies by examining the effect of increasing Pr level (for *x* = 0.1, 0.2 and 0.4) on the dynamic range of the refractive index modulation. We also introduce a tunable optical transmission device (shown in Figure 1A), that employs electrochemical oxygen titration [33] (“oxygen pumping” [34]), to vary the oxygen stoichiometry of the PCO thin film, and thus its optical properties. The voltage controlled transmission device, as we demonstrate, enables access to wide dynamic changes in optical transmittance, exhibiting modulations up to 75% in a 150 nm PCO thin film. Effects of PCO composition on device optical characteristics were analyzed both under steady state and switching (non-steady state) voltage conditions. Finally, *in-situ* video imaging of the transmission modulator with patterned top electrode, and the subsequent image analysis, show that the electrochemical titration approach enables spatially localized control of PCO’s optical properties and that the electrically programmed optical state can be made non-volatile by quenching to room temperature.

## 2 Experimental

PCO powders with *x* = 0.1, 0.2 and 0.4, denoted here by PCO10, PCO20 and PCO40, respectively, were synthesized by a solution combustion method [35], starting from Ce(NO_3_)_3_·6H_2_O and Pr(NO_3_)_3_·6H_2_O precursors (99.99%, Alfa Aesar). Stoichiometric amounts of nitrate-based cation precursors were dissolved in deionized water and citric acid was added as a fuel for combustion. The combustion, initiated by heating, lasted for 2 h, eventually yielding a reddish PCO powder that was subsequently calcined in a box furnace at 750 °C for 6 h in air. The obtained powder was used to prepare dense ceramic targets for pulsed laser deposition (PLD), following the procedure reported elsewhere [36].

For optical transmission measurements PCO20 and PCO40 thin films (190 ± 5 nm thick) were grown by PLD on single crystal sapphire substrates (c-cut, double side polished, 10 × 10 × 0.43 mm^3^), following the procedure described elsewhere [25]. The complex refractive indices of the PCO compositions were obtained, first by measuring the optical transmission in a custom-built spectrophotometer, capable of *in-situ* measurements at 500 °C in a controlled gas atmosphere. This was followed by fitting the total transmittance of the thin films, with the aid of the “RefFIT” program developed by Kuzmenko [37]. Further details regarding the experimental setup and the fitting process can be found elsewhere [25].

The electrochemical titration device, illustrated in Figure 1A, was prepared for thin film optical transmission modulation and switching measurements by providing means to modulate the oxygen vacancy concentration, or equivalently *δ*, and thereby the oxidation state of Pr in the film. It is composed of a four-layer planar structure, ITO/PCO/YSZ/Pt, where the indium tin oxide–ITO (∼70 nm thick) and PCO (∼150 nm thick) double layer was grown by PLD (same conditions as above) on yttria stabilized zirconia (YSZ) substrates (surface orientation is 100; double side polished; dimensions – 10 × 10 × 0.5 mm^3^). The top ITO layer is optically transparent and serves, concurrently, as an ionically blocking and electronically ohmic electrode. On the back side of the YSZ substrates, a Pt electrode (a grid of 5 µm wide lines spaced by 40 µm) was prepared by a standard photolithography process, prior to the oxide depositions. The Pt grid electrode passes ∼90% of the incident light and supplies electrons for the electrochemically driven oxygen exchange at the Pt-YSZ-gas triple phase boundary that sustains the oxygen ion’s current flow through the device.

*In-situ* video imaging of the electrochemical titration device, in a controlled temperature and gas environment, was performed inside a closed mini-chamber with parallel sapphire windows (Linkam stage, Scientific Instruments) with the aid of a long working distance optical microscope (Mitutoyo) in transmission mode. The device was back lit with a white light source, that for improved contrast, was cut-off below 550 nm by a short-pass filter (Thorlabs). Image analysis was performed on the unprocessed video frames (Adobe Photoshop software).

## 3 Results and discussion

### 3.1 Effect of PCO composition on the tunable range of optical properties

**Figure 2:**
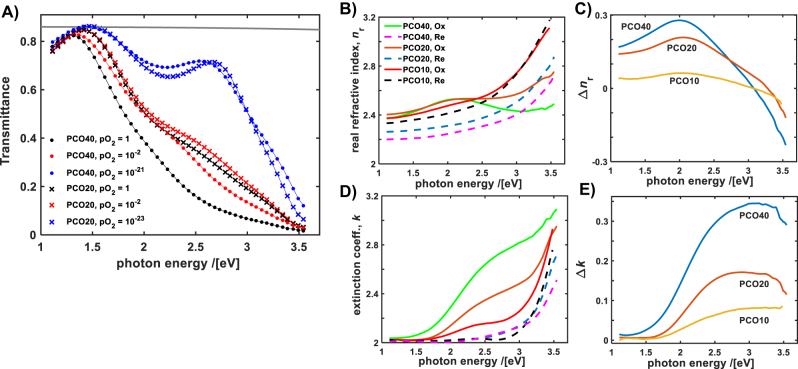
Dependence of PCO optical properties on the equilibrium *p*O_2_. (A) Optical transmittance of PCO20 (cross) and PCO40 (filled circle) at 500 °C, equilibrated at various *p*O_2_ (in bar units), as indicated in plot. Symbols – experimental data points, curves–modeled transmittance (see text for details). Bare Al_2_O_3_ substrate transmittance, ∼0.87, is shown as reference (grey curve). (B) *n*_r_ of the PCO compositions in the most reduced (dashed curves) and most oxidized states (solid curves). The PCO10 data was measured at 600 °C (adapted from reference [25]). (C) Change in the real part of the refractive index, Δ*n*_r_, between the most oxidized and reduced states in (B). (D) *k* – the extinction coefficient of PCO compositions corresponding to the *n*_r_ as shown in (B) (the same legend applies to the both plots) (E) Change in the extinction coefficient, Δ*k*, between the most oxidized and reduced states in (C).

An initial optical characterization of the PCO thin films, deposited onto sapphire substrates, was performed to investigate the dependence of the dynamic range of actively tunable optical properties (controlled by varying the oxygen activity in the PCO) on the Pr doping level, *x*. Figure 2A shows the optical transmittance of PCO20 and PCO40 thin films as a function of the equilibration *p*O_2_, measured *in-situ* at 500 °C. The covered *p*O_2_ range, from 1 to ∼10^−23^ bar (from pure O_2_ to 2000 ppm CO in CO_2_ mixture, respectively), spans nearly the whole redox range of the optically absorbing Pr^4+^ ion concentration, $\left[Pr^{4+}\right]$ . The latter varies with *p*O_2_, according to the defect model, as ${\left(x-\left[Pr^{4+}\right]\right)}^{3}{\left[Pr^{4+}\right]}^{-2}∼pO_{2}^{-1/2}$ , as detailed elsewhere [32, 38, 39]. Thus, the observed change in transmittance due to the change in oxidation state of the Pr cations between the near completely oxidized and near fully reduced state, is close to the theoretically possible maximum. Figure 2B shows the modeled (based on the total transmittance fitting), real part of the refractive index (*n*_r_) for PCO10 (from ref. [25], measured at 600 °C), PCO20 and PCO40 compositions in the two extremes, nearly completely oxidized and reduced states, c.f. solid and dashed lines. The change in *n*_r_ between the two oxidation states, Δ*n*_r_, increases with *x*, as shown in Figure 2C. In general, Δ*n*_r_ is wavelength dependent, exhibiting a local maximum near 2 eV (∼620 nm), more pronounced at higher Pr doping levels. The imaginary part of the refractive index, the extinction coefficient (*k*), is qualitatively similar in the PCO reduced state for all *x*, but significantly increases with *x* in the most oxidized state, as shown in Figure 2D. The corresponding Δ*k*, shown in Figure 2E, reveals that the increase is nearly proportional to *x*, in the 2–3.2 eV photon energy range. The latter indicates a weak or no dependence of the Pr^4+^ absorption cross section on *x*, with the effect of increased doping simply described by the Beer–Lambert absorption law. Comparison of the optical data presented in Figure 2B–E enables one to choose the working wavelength and optimize the tuned properties of the optical constants in the PCO compounds, depending on the tunable optical device requirements. For example, increasing the Pr level up to *x* = 0.4 provides a significant increase in the change of *n*_r_ with *p*O_2_ desired in e.g. phase optical actuators [28], however that comes at the price of higher corresponding optical losses, for photon energies above 2 eV.

### 3.2 Steady state optical tuning of the PCO–electrochemical titration device

**Figure 3:**
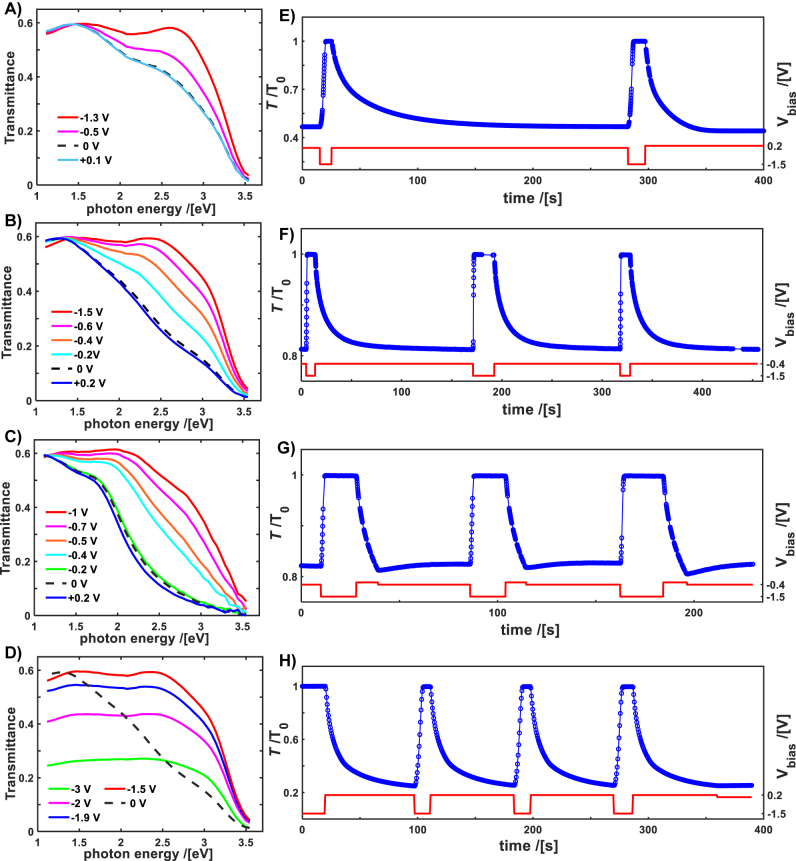
Actively tuned transmittance of the ITO/PCO/YSZ/Pt device, shown in Figure 1A, measured in a controlled environment in a *p*O_2_ of 1 bar, at 500 °C. The actively tunable PCO layer reduced and oxidized by electrochemical titration with voltage biases, indicated in legends or plotted, were applied on the top ITO electrode. Equilibrium transmittances, under voltage bias, are shown for (A) PCO10, (B) PCO20, (C) PCO40 compositions and for (D) PCO20 under extremely reducing conditions. (E) Time relaxation of the optical transmittance (blue circles, lines are a guide to the eye), normalized by a most transparent state, at photon energy 2.5 eV (495 nm) for the PCO20 device. Switching is controlled by voltage bias indicated on the same plot (red line). Full range switching for PCO20 is shown, where transmittance changes by more than 50%. Switching time constants are *τ*_on_ ∼ 3 s, *τ*_off_ ∼ 25 s. (F) Transmittance switching in PCO20 device, lower modulation depth, ∼15% instead of 50% shown in (E) (*τ*_on_ ∼ 1 s, *τ*_off_ ∼ 25 s) (G) Five times faster transmittance off switching, than in (G), achieved by, at first, voltage overshoot and then the set to the new value, c.f. with the applied voltage in (G) (*τ*_on_ ∼ 1 s, *τ*_off_ ∼ 5 s) (H) A full range transmittance switching for the PCO40 device exhibits a modulation of more than 75% percent (*τ*_on_ ∼ 5 s, *τ*_off_ ∼ 10 s).

Active tuning of the optical constants in the PCO compositions was studied here by applying electrochemical oxygen titration [33] (“oxygen pumping” [34]) that augments the previously reported chemical and electro-tuning methods [25]. We developed a transmission modulation device, see Figure 1A and experimental section for details, that enables precise electrochemical control over the oxygen vacancy concentration, likewise the oxygen activity, inside a PCO thin film supported on the YSZ solid oxygen ion electrolyte. Figure 3A–C shows the optical transmittance modulation in the electrochemical devices for different PCO compositions. The devices were operated and characterized at 500 °C to enable sufficiently rapid surface oxygen exchange at the Pt/YSZ interface and subsequent transport through the YSZ bulk. Most of the transmittance change is due to amplitude attenuation as the *k* changes significantly with *δ* (and thus $\left[Pr^{4+}\right]$ ) that spans from 0 to ∼0.5*x*, in the most oxidized and the reduced states, respectively (see also Figure 2E). Initially the PCO thin film is set to equilibrate at zero voltage bias (closed circuit conditions) in pure O_2_. The corresponding optical transmission in the initial state is shown as a dashed black curve in Figure 3. It is less transparent for devices with higher *x* (the active oxide film thickness is maintained at 150 ± 10 nm for all PCO compositions), as expected, following the trend of the corresponding extinctions coefficients, shown in Figure 2D. To modulate the optical transmittance, a constant voltage bias is applied across the device (Pt reference electrode grounded), leading to current flow until a new equilibrium is established between the oxygen activities in the PCO, $a_{O_{2},PCO}$ , and in the gas phase at the Pt reference electrode, $a_{O_{2},ref}$ , as measured across the YSZ electrolyte. According to the Nernst equation [40], $a_{O_{2},PCO}=a_{O_{2},ref}\times \exp\left(4qV_{\text{th}}/k_{\text{B}}T\right)$ , where *q* is the elementary electronic charge, *k*_B_ – Boltzmann constant, *T* – temperature in Kelvin, and *V*_th_ the Nernst open circuit voltage (note that $V_{\text{th}}<0\;\text{when}\;a_{O_{2},\text{ref}}>a_{O_{2},PCO}$ ). The applied voltage initially drives a Faradaic current through the ITO/PCO/YSZ/Pt structure, leading to oxygen ion accumulation/depletion in the PCO layer, given that the ITO electrode blocks oxygen exchange between the PCO film and the atmosphere. The current vanishes after a new equilibrium $a_{O_{2},PCO}$ is established [41]. Measuring the current transient provides information regarding the PCO oxidation state, *δ*, (and thus the optical properties) which according to Faraday’s law relates to the instantaneous current as d*δ* ∼ id*t* [33]. The bias voltages used here to modulate the optical properties are between positive 0.2 to negative 1.5 V. That yields, for a fixed surrounding reference activity ( $a_{O_{2},ref}$ ), corresponding to *p*O_2_ of 1 bar, $a_{O_{2},PCO}$ values corresponding to an effective *p*O_2_ range from ∼5 to 10^−39^ bar, enabling one to modulate the optical constants in PCO over the full range previously achieved by chemical means, c.f. Figures 2A and 3B–C. Application of a positive voltage bias (relative to the Pt back electrode) titrates oxygen ions (negatively charged) into the PCO layer, further decreasing the transmittance of the devices, as shown in Figure 3A–C. The device transmittance changes only by a few percent for a positive voltage bias, indicating that most of the optically active Pr ions are initially in the oxidized Pr^4+^ state, i.e. that *δ* is nearly zero for all compositions under these conditions. Under a negative applied voltage bias, oxygen ion depletion occurs within the PCO layer, progressing with the voltage bias increase. This leads to transmission increase, ultimately reaching the most transparent state (shown by red curves in Figure 3A–C) below a bias of −1 to −1.5 V, as most of the Pr ions are reduced to their 3+ state under those conditions. The device with the highest Pr level (PCO40), provides the maximum dynamical range of the optical change, c.f. Figure 3C and Figure 3B, consistent with the previously measured change in the optical constants shown in Figure 2B and Figure 2D.

An extremely reduced PCO20 device (biased below −1.5 V), on the contrary, shows a significant reverse in transmittance change with increasing voltage, though with a somewhat different wavelength dependence, as shown in Figure 3D. The latter observation can be explained by noting that while nearly all of the Pr ions are fixed in a Pr^3+^ state at these high magnitudes of negative applied bias, and thus cannot contribute to further decreases in transmittance, the majority cation Ce that remains stable as Ce^4+^ to much lower *p*O_2_ that Pr^4+^, does begin to reduce to Ce^3+^ [42, 43], while Zr^4+^ ions in YSZ begin to reduce to Zr^3+^, forming “black zirconia” [44]. The latter, reported previously to exhibit a strong absorption in the VIS, may be the reason behind the reverse in the transmittance change upon strong electrochemical reduction.

### 3.3 Non-steady state optical switching–electrochemical titration device

Figure 3E–H show repeated optical switching of the transmission tunable devices by a rectangular waveform voltage (red line shown on the plot, below the transmittance), under the same atmosphere and temperature conditions as before. At 495 nm (2.5 eV), the transmittance of the PCO20 device is modulated by more than a factor of 2, shown in Figure 3E, by switching voltage repeatedly between 0.2 to −1.5 V. The ON (from low to high transmittance) and the OFF (from high to low) switching time constants are, ∼3 and 25 s, respectively. The switching times are asymmetric, with ON leading to faster and OFF to slower transients (values shown in caption of Figure 3). The device’s switching dynamics, in general, can be attributed to different rate determining processes controlling the oxidation and reduction of the PCO film e.g. the transport of oxygen through the YSZ thick conductor and exchange of oxygen with the gas phase at the Pt/YSZ interface [27]. Redistribution of oxygen through thin film PCO deposited on a thick electrolyte has been demonstrated, previously, not to be a rate limiting process [45]. For the PCO20 device the ON switching time decreases for lower transmittance modulation, c.f. Figure 3E and F, as less oxygen needs to be shifted in and out of the film. The OFF switching time can be further decreased, by temporarily applying an “overshoot” voltage followed by a new set value, as shown in Figure 3G. The device containing the PCO40 thin film switches at a similar speed, c.f. Figure 3F and Figure 3H, but provides an enhanced contrast, factor of 3, between the ON and OFF states. In all studied cases, the optical transmittance change is reversible and shows high repeatability in the voltage programmed state. The proposed conceptual design of a reflective device illustrated in Figure 1B, employing electrochemical oxygen titration as well, should enable faster switching speed, as the ionic conductor there is a thin film with a correspondingly faster bulk transport time constant. Additionally, oxygen ions are stored in a solid oxygen “reservoir” layer that would replace the need for the likely slow gas-solid interface reaction by a much faster solid-solid interface reaction, where oxygen ions are transferred between the two layers directly [46].

### 3.4 *In-situ* video imaging of electrochemical switching device with patterned ITO surface

**Figure 4:**
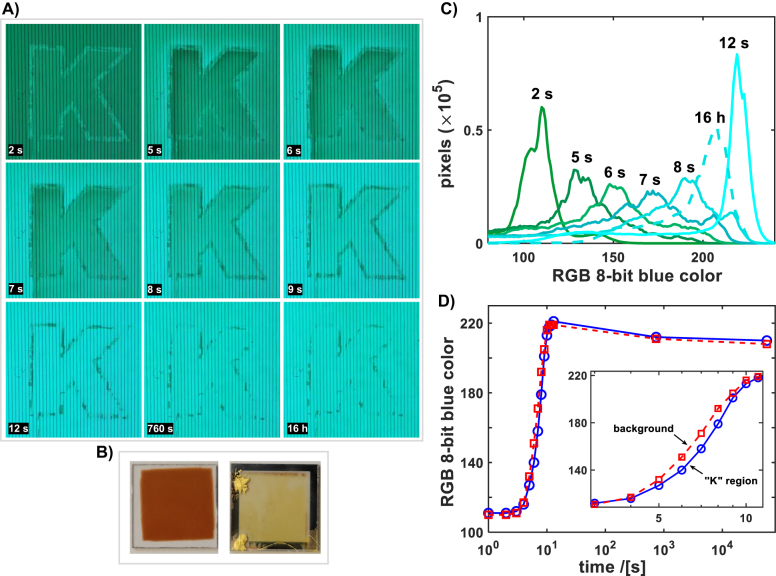
Patterned electrochemical switching device. (A) Selected video frames (time indicated on images) of patterned ITO/PCO40/YSZ/Pt device taken during electrochemical transmission switching and in the quenched state. The sides of each frame are 1.6 mm long with microscope focused on device’s back side; dark vertical lines are back Pt electrodes. The switching changes the transmission of the background area (around the “K” region) at first, and is then followed by oxygen ion redistribution within the whole non-patterned PCO40 layer, including the “K” region (see text for details). Last two frames show the quenched device in the most transparent (ON) state that remains non-volatile, beyond 16 h. (B) Images of the PCO40 thin film, left – on the Al_2_O_3_ substrate, equilibrated in O_2_ at 500 °C (oxidized) and quenched to room temperature in the highly absorbing state; right – PCO40 thin film inside the ITO/PCO/YSZ/Pt device, electrochemically tuned to the most transparent state (fully reduced), 16 h after quenching. Note easily distinguishable color contrast between the two thin films that remains non-volatile. (C) Quantitative analysis, blue color histogram (BCH), of change in the video frames shown in (A), (only the background region was included in the analysis). (D) Quantitative comparison analysis of the transmission switching of the biased background and the floating “K” regions in the PCO40 device. The dominant color tone (*y*-axis) was calculated at BCH center that evolves with time (see text for details). An inset shows a zoom-in to the time period when the switching transition occurs, highlighting a difference in the rate of the color change of the background (biased) and “K” (floating) regions.

For the experiments on spatially selective control of PCO optical properties, the ITO top layer of the ITO/PCO40/YSZ/Pt device used previously for electrochemical titration experiments, was patterned into two separate regions (ITO covered with patterned positive photoresist was etched with HCl acid). The middle region, with “K” letter shape, is separated from the rest of the ITO film (background area) by an etched groove, 50 µm wide. During switching experiments, shown in Figure 4, the background region was biased with respect to the Pt electrode on the back, while the K region was left floating (assuming that a current through an intact PCO40 layer is at least an order of magnitude less than through the ITO). Figure 4A shows a sequence of video frames taken *in-situ* during transmission switching of the device (from 2 to 12 s) and two additional frames (after 760 s and finally 16 h) taken in the thermally quenched-in state. At the initial equilibrium state, 0 V bias in *p*O_2_ of 1 bar at 500 °C, the whole device area has the same transmission (see Figure 4A). After a constant bias of −2 V is applied on the background region ITO electrode, its transmission gradually increases until a saturation level is reached after 12 s. In parallel, the transmission of the floating “K” region is also changing, but at slower rate, the change results due to non-perfect electrical insulation, for the present prototype device, e.g. current leaks from the PCO layer. Uneven change in the transmission of the biased versus floating regions, demonstrates that a spatial control of PCO optical constants can be achieved, in principle, by locally applied electrical fields. Eventually both regions reach the same steady state transmission after ∼12 s. Once the switching process is completed, the transmission of the devices increased by ∼3–7 times in the 2.3–3.2 eV range, as can be deduced from for the measurement taken on the same PCO40 device before the ITO layer was patterned, shown in Figure 3C. The thermally activated oxygen vacancies (or oxygen ions) transport in PCO [32, 38, 43] requires, on the one hand, elevated temperature for fast optical modulation, while on other hand, it enables implementation of non-volatility of the written state by thermal quenching. Here the non-volatility of a tuned transmission state is demonstrated by cooling the voltage biased device after switching, c.f. video frames in Figure 4A at 12 s, 760 s and 16 h, to room temperature. The transmission remains nearly the same following thermal quenching after 16 h, even though the device was kept in an atmosphere of pure O_2_, extremely far from the equilibrium state. Figure 4B visualizes the PCO40 thin film in the quenched initial state (here PCO40 on the Al_2_O_3_ substrate is shown), and the PCO40 device in the quenched final, electrochemically induced out-of-equilibrium state.

To quantify the spatial transmission changes of the device and their dynamics during the switching experiment, a color histogram analysis (using the blue component from the RGB color space, coded into the 0–255 range) of the video frames was implemented. Figure 4C shows evolution of the blue color histogram (BCH) during the transmission modulation, calculated for the background region only (see also Figure 4A). Initially, the PCO40 film is oxidized (more light absorbing) and the darker blue tones dominate (see in Figure 4A the video frame at 2 s), thus the corresponding BCH is centered around the 110 value, where it peaks. After the voltage bias is applied, the center value of BCH shifts to the lighter tones, though the change is not entirely homogeneous. Thus, the non-steady state BCHs are more smeared than those at the initial and final steady states (see Figure 4A). After 16 h, the peak position of the BCH of the quenched non-volatile state, stays close, but slightly below, the most transparent state of the device. Most of the observed change in color of the quenched state is due to a slight dependence of the PCO optical properties on temperature [25]. Analyzing a separate BCH of the background and the “K” regions, enables comparison of their switching dynamics, i.e. of the PCO40 under bias and the floating parts of the ITO electrode. The background and the “K” region BCHs were calculated from the video frames, e.g. the ones shown in Figure 4A. Next, Figure 4D shows the time evolution of the calculated BCH center values (approximated by the peak position) during and after the switching process. The analysis shows that following a rapid, ∼10 seconds, switching event, at extended times, both regions were quenched at nearly the same out-of-equilibrium state, while the region “K” was floating (see also discussion above). A zoom-in inset in Figure 4D, to the switching process time, shows that the biased background area switches faster to a new state, that becomes lighter, while the floating “K” region changes at slower rate, stays dark longer. Eventually the two patterned regions converge to the same transmittance at steady state (see also Figure 4A), due to the device design limitations, as explained above.

## 4 Conclusions

Tuning of the optical constant in the PCO compositions, with Pr doping levels as high as 0.4, was studied by varying the oxygen stoichiometry, *δ*, chemically, under oxidizing and reducing atmospheres, and by an electrochemical titration method operated in pure O_2_, at 500 °C. Calculating optical constants from the transmittance of the PCO thin films on sapphire substrate in the 1.1–3.5 eV range shows that increasing the Pr doping level extends the dynamic range of the refractive index modulation of both the real and the imaginary parts, while the latter scales nearly proportionally with *x*. Actively tunable transmission devices, based on electrochemical titration, were studied *in-situ*, at 500 °C, providing access to the equilibrium and non-steady state parameters related to transmission control of the PCO films, including the optical contrast, switching speed, reversibility and repeatability. Finally, the PCO40 device’s top ITO electrode was patterned to enable spatially selective optical tuning. *In-situ* video imaging during the electrochemical titration switching process, at elevated temperature, and following the quenching process, enabled quantitative analysis of the spatially selective optical tuning and non-volatility of the programmed, out-of-equilibrium, device state. The results of this study offer guidelines to achieve further enhancements in actively tunable transmission, and similar reflective devices, based on MIECs, e.g. as illustrated in Figure 1, with potential for future full color palette non-volatile display implementation.
